# Health education for reducing tobacco consumption in Shanghai based on the health versus financial framing effect: A randomized field study

**DOI:** 10.18332/tid/99610

**Published:** 2019-01-11

**Authors:** Lili Shi, Yuyang Cai, Hong Huang

**Affiliations:** 1 School of Public Health, Shanghai Jiao Tong University School of Medicine, Shanghai, China

**Keywords:** tobacco control, health education, framing effect

## Abstract

**INTRODUCTION:**

In China, the regular use of tobacco by much of the population has brought great losses in both health and economy. This study used the framing-effect theory to explore people’s preferences regarding message-framing for education about financial and health-related gains associated with smoking cessation in different areas of Shanghai, China. In particular, it attempts first to identify any influencing factors and offers some suggestions to improve the efficiency of education efforts regarding tobacco control.

**METHODS:**

A message-framing field study was conducted in three districts in Shanghai that represent low-income, middle-income and high-income areas between June and August 2015, respectively. This study examines leaflets with a different message-framing (Health-Financial Framework) to ascertain the preferences of participants at the different locations. A total of 4650 participants were recruited and asked to complete a questionnaire. The z-test, chi-squared test and logistic regression analysis were used for statistical analysis.

**RESULTS:**

In low-income areas, participants were more inclined towards financial rather than health message-framing, especially in financial and health oriented locations. In the middle-income areas, participants preferred health message-framing, especially at hospital locations. In the high-income areas, health message-framing was preferred in health and neutral locations in comparison with financial locations. The type of area, location and household monthly income have an impact on participants’ preference for message-framing.

**CONCLUSIONS:**

Three suggestions are proposed for tobacco control health education in China: 1) Combine health education information design with the framing-effect theory to improve the effectiveness of information content, 2) Considering the impact of different types of area and location, different tobacco control health education strategies should be adopted to improve the efficiency of publicity, and 3) Targeted and personalized tobacco control health education strategies should be implemented among different audiences.

## INTRODUCTION

The use of tobacco has brought great losses in both public health and economic prosperity in China. In 2013, the number of cigarettes consumed in China was equal to the combined consumption of low- and middle-income countries^1^. By the end of 2015, the number of annual deaths caused by smoking in China had exceeded 1.4 million^1^, and it is expected to exceed 3 million per year by 2050. Since China is a member of the Framework Convention on Tobacco Control (FCTC) of the World Health Organization (WHO), a series of measures and strategies intended to curb consumption have been formulated and implemented^2^. However, people’s awareness of the harms of tobacco remains poor; thus, attempts to increase such awareness need to be further strengthened^3,4^.

Many studies confirm that the framing-effect theory is both applicable and highly useful when researching public education for smoking reduction. The term ‘framing effect’ refers to the change of preference in a given choice caused by adjustments made in the presentation of information^5^. The framing effect was proposed by Tversky and Kahneman in 1981. Rothman and Salovey found that the positive framework was more convincing in promoting smoking cessation^6^,^7^; the negative framework did better in the disease detection behaviour^8,9^. Regarding education to curb tobacco consumption, Toll et al.^10^ found in a clinical trial in 2008 that the smoking cessation rate was higher and the relapse interval was longer in women smokers who had received positive framing than those who had received negative framing. The results of a randomized trial in a high school showed that under the negative framework, adolescents were more concerned about elderly respiratory disease information (p<0.05)^11^. Bresnahan et al.^12^ claimed that compared to the self-targeted messages, students were more susceptible to positive messages; negative framed messages had little impact on smoking teenagers and non-smoking teenagers. Mollen and Engelen^13^ found that positive framed messages showed stronger effects on intentions to quit smoking than negative framed messages when participants were concerned about short-term outcomes.

Sindelar and O’Malley^14^ did a randomized field study on financial versus health motivations to quit smoking in 2009 in New Haven, USA. They designed two types of leaflets, expressing either financial or health-related benefits of smoking cessation in order to encourage readers to quit smoking. These leaflets were placed in check cashing centres, clinics, and grocery stores, which represent financial, health-related, and neutral types of locations, respectively. They then calculated the number of leaflets taken away from each location and compared the differences between each location. They used the selection of the leaflet as a proxy for interest in quitting smoking, understanding that interest is a necessary step that often serves as a proxy for both motivation and action. The results of their study indicated that in low-income areas, finance-related messages could generate more interest in quitting smoking than health-related messages. Additionally, finance-related messages could also be more powerful in commercial settings. However, the results of their study might not be generalizable across different geographical regions. Also, the study neither focused on the rate at which participants took the leaflets nor determined what factors influenced their choice to take the leaflet.

In China, research related to the reduction of tobacco consumption was always of high importance. However, no studies to date have determined whether framing effects noted in other regions may hold for China as well. Therefore, in this study we replicate for Shanghai the Sindelar and O’Malley study; specifically focusing on determining the differences in framing preferences—between finance-related and health-related—in low, middle and high income areas to prove the effect of message-framing. After presenting our results, we conclude by offering suggestions on how to improve the efficiency of education efforts designed to curb tobacco consumption in the Chinese context.

## METHODS

Between June and August in 2015, tobacco control education leaflets with both finance-related and health-related framing were placed in low, middle, and high-income areas in Shanghai in three different settings—banks, hospitals, and residential areas. The two types of leaflets were placed on the exhibition shelves on-site, marked ‘Take one leaflet only’, in each setting. The number of each type of leaflet that was taken from each setting at each location was recorded. Each time a person picked up a leaflet, they were asked by the on-site research staff to fill in a questionnaire if they agreed. Every working day from June to August 2015, the research staff worked on-site during open hours (9 am to 5 pm) at the banks, hospitals, and residential areas. They asked every person who picked up a leaflet if they agreed to participate in the interview. The leaflet was allowed to be taken away even when people did not wish to fill in the questionnaire. When 500 questionnaires were filled in at a location, that location was closed.

### Message framing

Two sets of leaflets were produced with visual and written messages to motivate readers to quit smoking. One of them emphasized the benefits of smoking cessation for one’s health, and the other emphasized the financial benefits. Messages were gain-framed— i.e. they focused on the positive benefits that would result over time if someone quit smoking. The health leaflets were titled ‘Quitting smoking brings health to you’, and described the health improvements over one week, one month, and one year. On the back side of the leaflets, further long-term health benefits were described. Similarly, the financial leaflets were titled ‘Quitting smoking brings wealth to you’ and described the financial gains over one week, one month, and one year. The other side of the leaflets gave further details about how much money could be saved in the long term if participants quit smoking, according to each brand’s pricing schemes.

**Figure 1:**
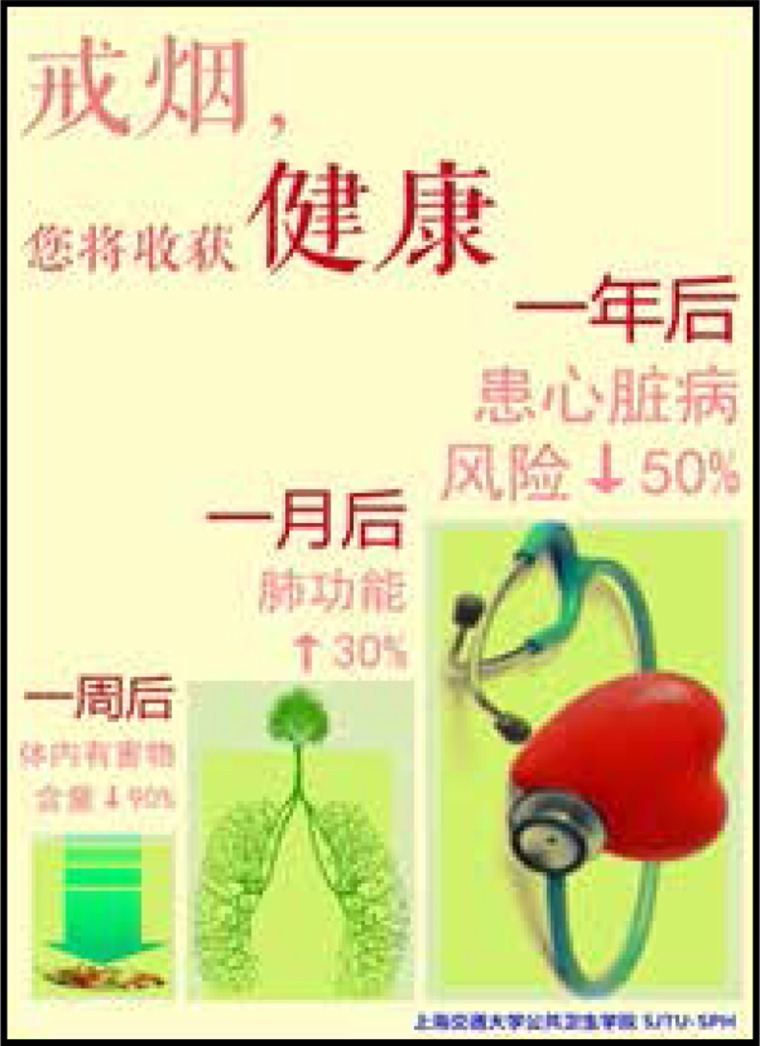
Health framing leaflet (front)

**Figure 2:**
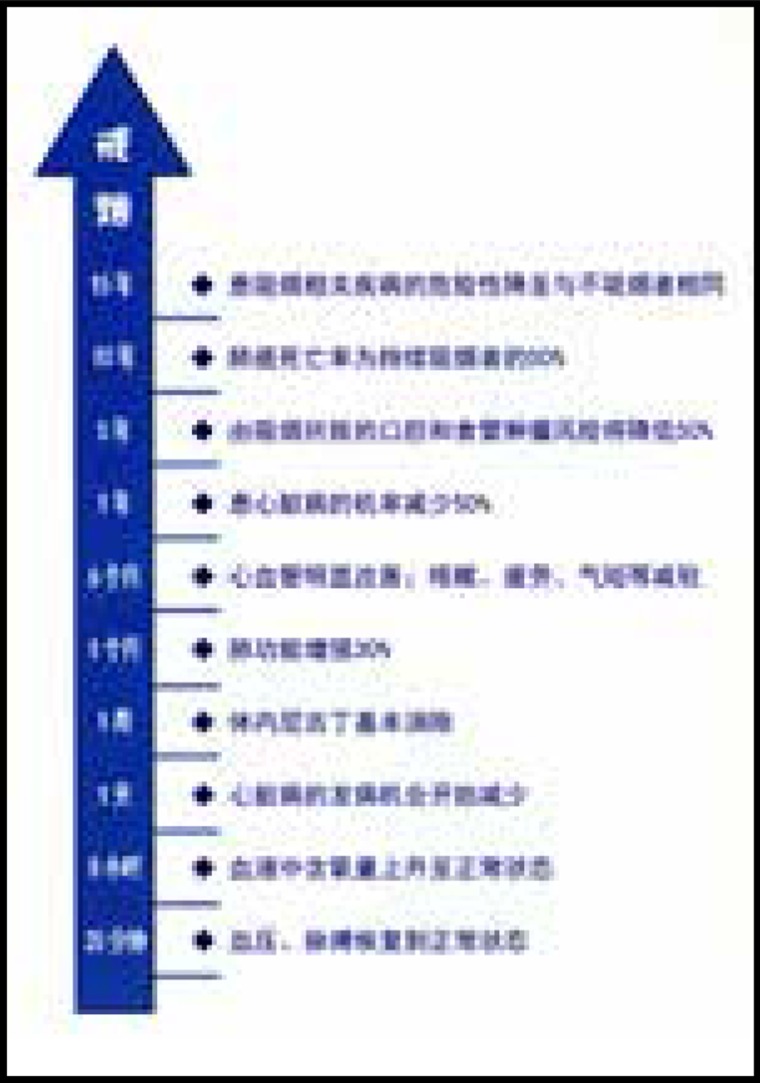
Health framing leaflet (back)

**Figure 3:**
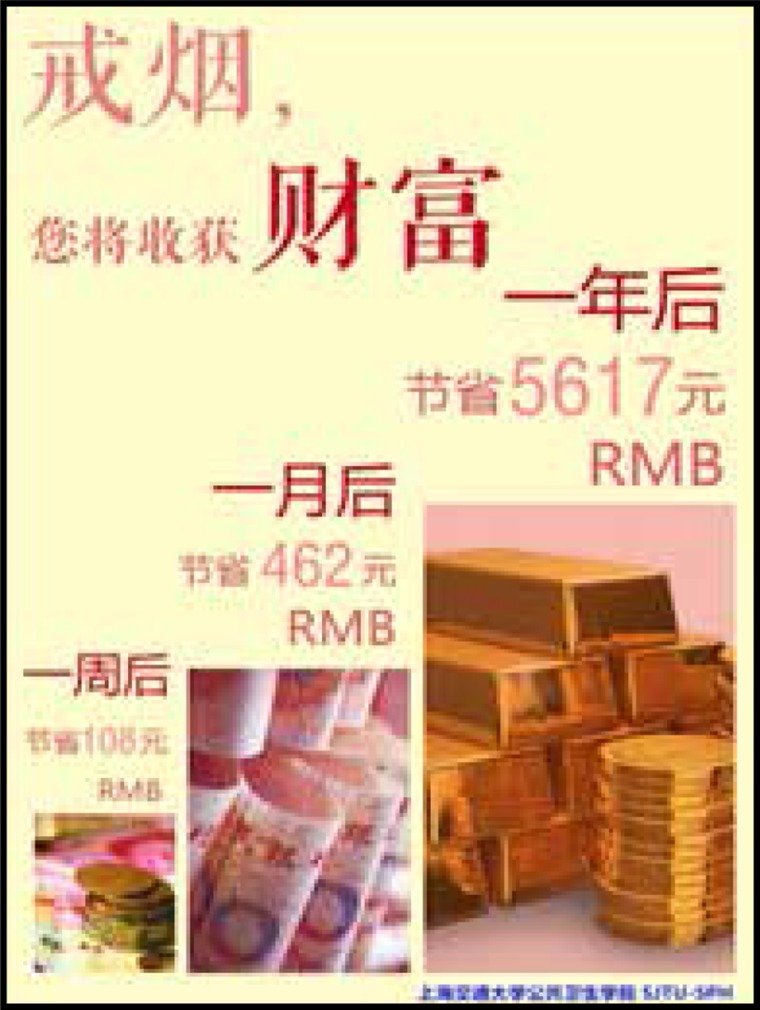
Finance framing leaflet (front)

**Figure 4:**
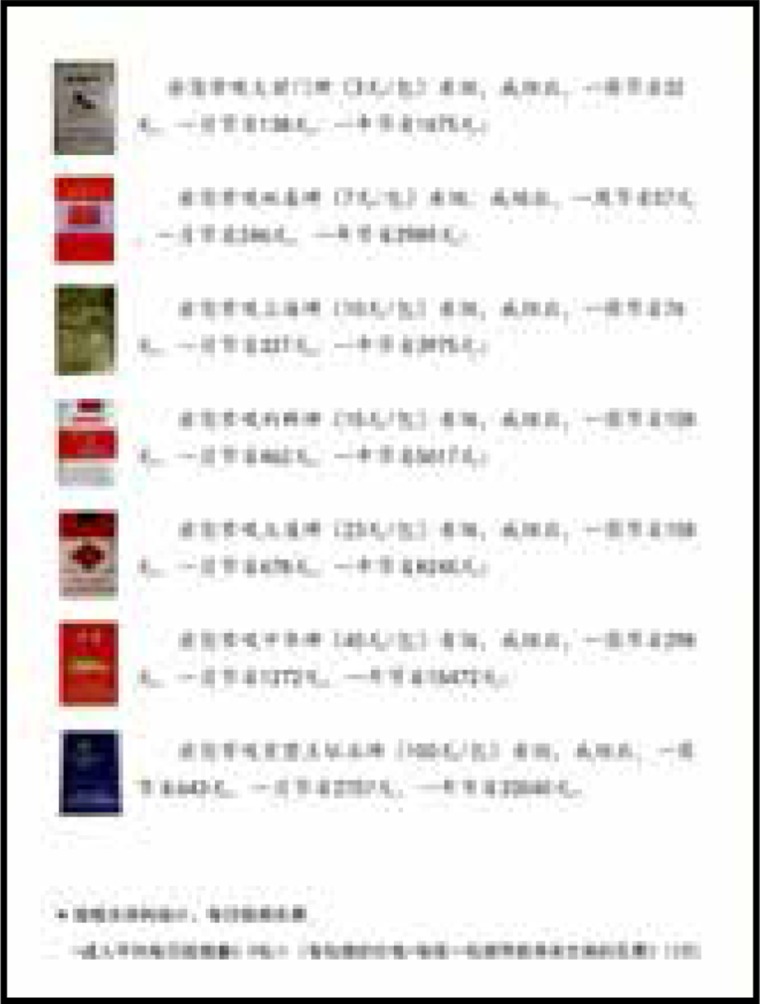
Finance framing leaflet (back)

The content of health education information on tobacco control used in this experiment is based on scientific evidence. The background color of the pages is the same warm yellow. The pictures also have obvious health or financial characteristics, reducing the interference of additional information. The content of the pages referred to Yale University’s research and refined according to science, as shown in Figures 1–4.

### Questionnaire

The questionnaire had four parts, including 17 questions, which take about 5 minutes to be completed. The first part asked about the respondent’s preference for message framework—either financial or health-related. The second part collected sociodemographic information of the respondents. The third part asked about their current smoking status. The last part asked about their smoking cessation experience, which could remain unanswered if the respondent did not smoke. The questionnaire was based on the Tobacco Investigation Questionnaire (Second Edition) of the Global Adult Tobacco Survey (GATS) developed by WHO^12^ (questionnaire is given as a Supplementary file). Both the validity and reliability of the questionnaire were analyzed during the study. The questionnaire was considered reasonable and acceptable by the respondents.

### Sites and sample populations

The field survey targeted participants over 15 years old. Based on the 2015 Shanghai housing price statistics, the 2014 GDP and per capita GDP ranking of Shanghai districts, and the 2015 household per capita disposable income, 17 districts and counties in Shanghai were divided into high-income, middle-income, and low-income areas. One district was randomly selected from each income area. In the three selected districts, Jing An District (high-income areas), Chang Ning District (middle-income areas) and Pu Dong suburbs (low-income areas), one hospital for the health location, one bank for the financial location, and one residential area (neutral location) were randomly selected from each district, and we proceeded to conduct the on-site study and face-to-face questionnaire interviews in each of the 9 sites. Before the start of the study, the on-site research staff received standardized training on how to appropriately monitor the leaflet locations and conduct the face-to-face interviews. In each location, staff placed an equal number of the financial and health leaflets on the exhibition shelves during the opening hours, marked with ‘Take one leaflet only’. The two types of leaflets were placed adjacent to each other, and anyone who entered the place was free to choose one without any promotion. Anyone who took a leaflet would become a potential respondent automatically for the interview. The staff would inquire whether the person was over 15 years old and willing to participate in the study when they picked the leaflet. After giving consent, the participants would be asked to complete the questionnaire. Every day, the on-site staff recorded the number of leaflets remaining and restocked the shelves.

### Statistical analysis

Statistical software SPSS19.0 was used for data analysis. Z-test and a chi-squared tests were used to test the validity of the data and the difference of the test results. Binary logistic regression analysis was used to test the influencing factors for the respondents’ preference, adjusting for age, gender, and occupation.

## RESULTS

### Sample characteristics

Throughout three months, 4912 leaflets were picked up —2540 were health (51.7%) and 2372 were financial (48.3%). There were 4650 (94.6%) questionnaires filled in (262 people refused the interview) —1650 in the high-income area (35.5%), 1500 in the middle-income area (32.3%) and 1500 in the low-income area (32.3%). According to location types, a total of 1550 leaflets were picked up in the health, financial and neutral locations, respectively. The sociodemographic information of the 4650 respondents is shown in Table 1. The average monthly household income in the high-income, middle-income, and low-income areas was 9631.7, 5781.3 and 3283.1 Yuan, respectively. The differences between the three groups were statistically significant (p<0.001). Moreover, the average monthly household income of respondents in the three districts corresponded well with the definition of Shanghai’s high-income, middle-income, and low-income areas.

**Table 1:** Sociodemographic characteristics of participants in the field study, Shanghai, China, 2015

	*Area*			*Total*	*P*
	*High-income*	*Middle-income*	*Low-income*		
**Location type**					
Health	550	500	500	1550	
Financial	550	500	500	1550	1.000
Neutral	550	500	500	1550	
**Age, mean (years)**	49.48	47.18	52.70	49.78	<0.001
**Gender**					
Male	897	694	668	2259	<0.001
Female	753	806	832	2391	
**Education level**					
Primary school and lower	76	55	391	522	
Secondary school	862	724	866	2452	<0.001
Tertiary and higher	673	720	225	1618	
Unknown	39	1	18	58	
**Household monthly income (Yuan)**					
<4001	334	765	1232	2331	
4001-7000	438	470	219	1127	
7001-10000	445	170	27	642	<0.001
>10000	217	95	22	334	
Unknown	216	0	0	216	
**Health selfevaluation**					
Good	903	677	933	2513	
Fair	640	735	508	1883	<0.001
Poor	80	57	34	171	
Unknown	27	31	25	83	
**Smoking status**					
Yes (>4 times per week)	452	342	479	1273	
No	1085	1156	1019	3260	<0.001
Unknown	113	2	2	117	
Smoking amount (cigs/day)*					
<11	187	116	99	402	
11-12	234	157	285	676	
21-30	11	40	32	83	
31-40	10	19	27	56	<0.001
>40	3	3	2	8	
Unknown	7	7	34	48	
**Smoking expense (Yuan/ month)**										
<101	22	11	18	51	
101-200	42	44	71	157	
201-300	81	92	143	316	
301-400	98	48	74	220	<0.001
401-500	107	67	44	218	
>500	84	74	93	251	
Unknown	18	6	36	60	
**Smoking cessation desire**										
Yes	268	182	218	668	
No	141	146	98	385	0.001
Unknown	43	14	163	220	
**Tried to quit smoking**										
Yes	229	152	189	570	
No	151	174	127	452	<0.001
Unknown	72	16	163	251	
**Reasons of smoking cessation**										
Family	242	75	151	468	
Cessation promotion	7	22	30	59	
Doctor's advice	12	19	18	49	<0.001
Desire for autonomy	39	57	36	132	
Unknown	152	169	244	565	
**Motivation to quit smoking**										
Personal health	228	133	129	490	
Family's health	64	30	74	168	
Financial stress caused by disease	3	2	7	12	<0.001
Other financial stress	3	2	9	14	
Others	6	6	1	13	
Unknown	148	169	259	576	

* cigs: cigarettes

### Message-framing preferences in each location and setting

Overall, more health leaflets (51.36%) were picked up than financial leaflets (48.64%), which was found to be a significant difference (p<0.05). According to Table 2, more health-related leaflets were picked up than financial leaflets in the high-income (56.1%) and middle-income (50.3%) areas. In contrast, more financial leaflets (52.8%) were chosen than health leaflets (47.2%) in the low-income areas. Difference between pickup rates of financial and health leaflets was significant across areas comparing: high-income areas versus low-income areas; and high-income versus middle-income areas. However, in the high-income areas, participants preferred the health messages over the financial messages. Finally, although the pickup rate of the health leaflets in the middle-income areas was higher than that of the financial leaflets, as with the high-income areas, the difference between the pickup rates was not significant.

**Table 2:** Number of leaflets picked up by financial and health message type in Shanghai, China, 2015

*Message type*	*Financial messages*	*Health messages*	*Total messages*	*Financial messaging as a % of the total (response variable)*	*Statistical level of the test of difference: p-value*	
					*Difference in each area*	*Difference across areas*
High-income area	725	925	1650	43.9	-4.92	High-income area against Middle-income: p=0.001 Low-income: p<0.001
Middle-income area	745	755	1500	49.7	-0.26	
Low-income area	792	708	1500	52.8	2.17	Low-income area against Middle-income: n/s High-income: p<0.001

According to the location type, participants who picked up leaflets at the hospitals (health-related locations) (55.1%) and residential areas (neutral locations) (53.6%) were more concerned about health, while those who picked up the leaflets at banks (financial locations) were more concerned with finance (54.6%) (Table 3).

**Table 3:** Number of leaflets picked up by location type in Shanghai, China, 2015

*Location type*	*Financial messages*	*Health messages*	*Total messages*	*Financial messaging as a % of the total (response variable)*	*Statistical level of the test of difference: p-value*	
					*Difference between financial vs health messages at each location*	*Difference between financial vs health messages across locations*
Financial: Bank	847	703	1550	54.6	3.66	Financial against Health: 0.000 Neutral: 0.000
Health: Hospital	696	854	1550	44.9	-4.01	Health against Financial: 0.000 Neutral: 0.407
Neutral: Residential	719	831	1550	46.4	-2.84	

For the low-income areas, participants preferred the financial leaflets over the health leaflets in both the financial locations (58%) and health locations (54%). For the middle-income areas, more participants took financial leaflets than health leaflets at both the bank and residential areas. However, health-related leaflets were more attractive than financial ones at the hospital. Finally, in the high-income areas, in contrast to the low-income areas, more health leaflets were picked up in both health and neutral sites than financial leaflets. However, at the banks, more financial leaflets were selected (Table 4).

**Table 4:** Number of leaflets picked up by location type in low-income, middle-income, and high-income areas in Shanghai, China, 2015

*Location type*	*Financial messages*	*Health messages*	*Total messages*	*Financial messaging as a % of the total (response variable)*	*Statistical level of the test of difference: p value*	
					*Difference financial vs health messages at each location*	*Difference financial vs health messages across locations*
**Low-income area**						
Financial: Bank	290	210	500	58.0	0.000	Financial against Health: 0.001 Neutral: 0.001
Health: Hospital	270	230	500	54.0	0.000	Health against Financial: 0.001 Neutral: 0.559
Neutral: Residential	232	268	500	46.0	0.000	
Total	792	708	1500	52.8	0.000	
**Middle-income area**						
Financial: Bank	267	233	500	53.4	0.000	Financial against Health: 0.001 Neutral: insignificant at standard levels
Health: Hospital	215	285	500	43.0	0.000	Health against Financial: 0.001 Neutral: 0.003
Neutral: Residential	263	237	500	52.6	0.000	
Total	745	755	1500	49.7	0.000	
**High-income area**						
Financial: Bank	290	260	550	52.7	0.000	Financial against Health: 0.002 Neutral: 0.000
Health: Hospital	211	339	550	38.4	0.000	Health against Financial: 0.002 Neutral: 0.090
Neutral: Residential	224	326	550	40.7	0.000	
Total	725	925	1650	43.9	0.000	

In the binary logistic regression analysis (Table 5), after adjusting for age, gender, and occupation, the results showed that there was a clear link between the location types and the preferences of the participants. Participants from the high-income areas were more likely to choose the health framework. In the financial locations, participants preferred the financial framework. Finally, after adjustment, the impact of household per capita monthly income and occupation on preferences for information frameworks was found to be rather weak (Table 5).

**Table 5:** Association between factors and framing preference in Shanghai, China, 2015

	*Unadjusted*			*Adjusted*		
	*Crude OR*	*95% Cl*	*p*	*AOR*	*95% Cl*	*p*
**Area**			**<0.001**			**0.014**
High-income	-	-	-	-	-	-
Middle-income	0.794	0.690-0.914	0.001	0.831	0.710-0.973	0.022
Low-income	0.882	0.609-0.806	<0.001	0.780	0.656-0.982	0.005
**Location type**			**<0.001**			**<0.001**
Health	-	-	-	-	-	-
Financial	0.676	0.587-0.779	<0.001	0.671	0.576-0.781	<0.001
Neutral	0.942	0.818-1.085	0.407	0.933	0.809-1.077	0.346
**Household monthly income (Yuan)**			**<0.001**			**0.003**
<4001	-	-	-	-	-	-
4001-7000	1.464	1.269-1.690	<0.001	1.334	1.142-1.559	<0.001
7001-10000	1.268	1.064-1.510	0.008	1.092	0.893-1.335	0.391
>10000	1.209	0.961-1.521	0.106	1.057	0.818-1.365	0.673

Binary logistic regression analysis was used to test the influencing factors of the respondents' preference behavior adjusting for age, gender, and occupation.

## DISCUSSION

Overall, this study shows that the impact of educational materials designed to encourage smoking cessation is indeed influenced by the types of frames that are used to convey the message. In high-income areas, participants prefer the Health Framework, in low-income areas participants prefer the Financial Framework, and in middle-income areas, participants have no clear preference for either the Health or Financial Framework.

In health-related and neutral locations, participants were more inclined to choose messages that were within the health-related framework; in financial locations, participants were more inclined to choose those that emphasize financial gain. One possible reason why people in China are more interested in the health-related framework could be that the cost of consuming tobacco in China is relatively low. In 2015, people in Shanghai spent only about $1.08 (exchange rate 6.5 Yuan to a US$) on a cigarette pack. According to the income per capita of the year, for a pack-a-day smoker, the proportion of tobacco expense to income per capita is 5.09% (for income $58908) or 3.58% (for income $10964). Thus, the financial gain of quitting smoking is less, and people are therefore not as strongly affected by that type of motivation.

According to the different income level areas and types of location, in the high-income areas, participants tended to choose the health framework in all locations; in the low-income areas participants tended to choose the financial framework in both the health and finance-related locations and the health framework in the neutral locations. Finally, in the middle-income areas, participants were more interested in the health framework at the health-related locations and, similarly, preferred the finance framework at the finance-related locations. This is perhaps because participants in high-income areas have higher household monthly incomes, and their health concerns are more significant than financial conditions. While the financial framework details merely how much money one could save, the health framework offers more subjective or personal benefits, such as longer life, better health, and more. However, participants in low-income areas tend to be more concerned about their financial conditions than their health status. In neutral locations, the respondents’ tendency was to choose the health framework, which is different from the USA result.

In addition to the type of area and location, occupation also affected the framework preference of participants. Compared to other occupations, participants engaged in agriculture, forestry, animal husbandry and the fishery industry preferred financial information. Participants who intend to quit smoking mainly due to ‘family discouragement’ or ‘self-motivation’ preferred health information. Participants with a household per capita monthly income below 4000 Yuan were more inclined to choose the financial framework. Also, framework preference was not affected by individual factors such as gender, age, personal income, smoking status, and smoking cessation experience. According to the framing-effect theory, gender, age, and smoking status typically are only related to the Loss-Benefit Framework. However, this study is a one-way framework based on the Benefit Framework, so it is understandable that gender had no relationship with the health-financial framework.

Overall, this study has found some results that differ from the Sindelar and O’Malley study in the USA, especially regarding low-income areas and neutral locations. These divergences may be due to the differences between the two studies and differences between the two countries in tobacco prices and health education strategies. First, in the US study, grocery stores were used to represent the neutral location, but by comparison, grocery stores are more ‘financial’ compared to residential areas. This may lead participants to choose the financial framework. Second, the tobacco price in Shanghai is lower than in Connecticut, which makes participants potentially care more about information related to health rather than finance. Last, participants of low-income areas preference for health information in neutral locations might vary according to years of education about the health harms of smoking, which was the main content of tobacco control education in China.

Results notwithstanding, this study does have certain limitations. First, the study only focuses on testing the usefulness of the Health-Financial Framework for tobacco-related education messages in China, and thus no other frameworks are involved. Second, the study did not consider the situation where people may choose both types of leaflets, which means the two message framings have no significant difference in affecting people’s performance. Third, the study used only the Benefit framework and thus can offer no information regarding the Loss framework in the Chinese context. However, according to theoretical research, the Loss Framework may not have a significant effect on tobacco control information. Fourth, due to the incomplete experimental data from Yale University, it is difficult to compare differences between the two studies, in China and the United States, and the influencing factors. Finally, this study was unable to verify any potential causal associations between the frameworks used to present messages to encourage smoking cessation, any improvement in awareness of the harms of tobacco, and any consequent changes in behavior.

## CONCLUSIONS

Based on the results, the following suggestions are proposed for tobacco control health education in China. First, health education information should be designed in conjunction with the framing effect theory to improve the effectiveness of the message. Different behavior objectives, framework types, information presentation and description, time and background and other factors must all be taken into account when deciding how to frame the message at hand. The individual factors of the receiver need to be fully considered in order to increase the receiver’s sense of involvement with the message. Second, considering the interactions noted between different areas and types of locations and the framework preferences, different tobacco control health education strategies should be adopted in different areas and locations to improve the efficiency of publicity. Third, targeted, personalized tobacco control health education strategies should be developed for different sociodemographic populations, e.g. pregnant women and their spouses. Furthermore, tobacco control health education can also be further analyzed from the perspective of family relations.

Research on the effect of different framing strategies on the strength and efficiency of tobacco control health education strategies in China has just begun. Future research should expand upon the present study to include other types of frameworks and different target groups, such as adolescents. Finally, although studies both in China and abroad, have shown that framing strategies do affect tobacco control health education, the mechanism by which it has this effect is still unclear.
