# Supplementary material for: Health education for reducing tobacco consumption in Shanghai based on the health versus financial framing effect: A randomized field study
Source: Tob Induc Dis. 2019 Jan 11;17:03. doi: 10.18332/tid/99610 (PMC6752000; doi:10.18332/tid/99610)
Supplement: Supplementary file 1 [file TID-17-03-s1.pdf]

Questionnaire on Information Framework Effect of Tobacco Control Health Education

Questionnaire Informed Consent

Hello!

We are the research team of Shanghai Patriotic Health Campaign Committee Office. We invite you to participate in our questionnaire survey. The survey is anonymous and there are no right or wrong answers to these questions. The survey will take you about 5 minutes. After the survey, there has a small gift for you. Your answers will be strictly confidential. We hope to get your support and cooperation. Thank you!

Shanghai Patriotic Health Campaign Committee Office

Questionnaire

Number of questionnaire □□□□□

PART 1: Choice of leaflet

□A1 Please look at the two leaflets we have shown you and choose one that you are interested in and willing to take away (only one is permitted):

① Health ② Economy

□A2 The reason for choosing this leaflet is：

① It’s the right answer ② I am interested in the health information on the leaflet ③ I am interested in the economy information on the leaflet

PART 2: Demographic information

□B1 Sex：①Male ②Female

□B2 Age: ______ years old

□B3 The highest academic qualification you've gotten？

①Primary School and Under ②Secondary School ③ Tertiary and Above

□B4 Occupation:

①Agricultural, forestry, animal husbandry, fishery and aquaculture production personnel
②Production and transportation equipment operators and related personnel

③Commercial and service personnel ④Personnel in charge of state organs, party and mass organizations, enterprises or public institutions ⑤Staff of government, enterprises or public institutions ⑥Professional and technical personnel ⑦Army People ⑧Other workers ⑨Students ⑩Unemployed ⑪Retired persons

□B5 The average monthly income of **you** in the last 1 year is________ Yuan.

□B6 The average monthly income of **your family** in the last 1 year is________ Yuan.

□B7 How do you feel about your health? ① Good ② Fair ③ Poor

PART 3: Smoking status

□C1 Do you smoke (>=4 times per week) ? ① Yes（go to C2） ②No（interview is completed)

□C2 How many cigarettes do you smoke every day? _________

□C3 What's the cigarette brand you commonly used?_________________

□C4 How much do you spend on smoking every month? ____________________

PART 4: Smoking cessation experience

☐ D1 Do you have willing to quit smoking? ① Yes ②No

☐ D2 Have you ever tried to quit smoking? ① Yes ②No

☐ D3 The main reason why you quit smoking is:

①dissuasion from family members ②smoking cessation promotion ③doctor's advice ④desire for autonomy

☐ D4 What's your main motivation to quit smoking? ①your own health ②your family health ③economic pressure caused by illness ④economic pressures from other reason ⑤others ________________

Thank you for participating in our interview. A small gift is given for your cooperation.

Time of investigation：2015Year ___ Month ___ Day

Location number of investigation：__________________

Signature of research staff：_________________________

© 2019 Shi L.
